# *Helicobacter* and the Potential Role in Neurological Disorders: There Is More Than *Helicobacter pylori*

**DOI:** 10.3389/fimmu.2020.584165

**Published:** 2021-01-28

**Authors:** Nina Gorlé, Eva Bauwens, Freddy Haesebrouck, Annemieke Smet, Roosmarijn E. Vandenbroucke

**Affiliations:** ^1^ VIB Center for Inflammation Research, Ghent, Belgium; ^2^ Department of Biomedical Molecular Biology, Faculty of Sciences, Ghent University, Ghent, Belgium; ^3^ Department of Pathology, Bacteriology and Avian Diseases, Faculty of Veterinary Medicine, Ghent University, Merelbeke, Belgium; ^4^ Laboratory of Experimental Medicine and Pediatrics, Faculty of Medicine and Health Sciences, University of Antwerp, Antwerp, Belgium

**Keywords:** *Helicobacter pylori*, *Helicobacter suis*, microbiome–gut–brain axis, gut microbiota, neurological disorders

## Abstract

Trillions of symbiotic microbial cells colonize our body, of which the larger part is present in the human gut. These microbes play an essential role in our health and a shift in the microbiome is linked to several diseases. Recent studies also suggest a link between changes in gut microbiota and neurological disorders. Gut microbiota can communicate with the brain *via* several routes, together called the microbiome–gut–brain axis: the neuronal route, the endocrine route, the metabolic route and the immunological route. *Helicobacter* is a genus of Gram-negative bacteria colonizing the stomach, intestine and liver. Several papers show the role of *H. pylori* in the development and progression of neurological disorders, while hardly anything is known about other *Helicobacter* species and the brain. We recently reported a high prevalence of *H. suis* in patients with Parkinson’s disease and showed an effect of a gastric *H. suis* infection on the mouse brain homeostasis. Here, we discuss the potential role of *H. suis* in neurological disorders and how it may affect the brain *via* the microbiome–gut–brain axis.

## Introduction

The human microbiota contains trillions of symbiotic microbial cells that live in and on our body of which the vast majority are present in the human gut (1–4). These commensal microbes perform several functions essential to our health and survival, including food digestion (5, 6), activation of certain drugs (4), prevention of infections (7–9), and they might play a role in the maturation of our immune system (10, 11).

Already for a few decades, changes in the gastrointestinal microbiota have been associated with a wide range of health problems including rheumatoid arthritis, inflammatory bowel diseases, asthma, and cancer, et cetera (12–17). Moreover, it has been shown that gastrointestinal changes are able to influence neurological disorders such as depression, anxiety, Alzheimer’s disease, Parkinson’s disease, and multiple sclerosis (MS) (18–29). Recently, it became clear that the gut microbiome can signal to the brain *via* several pathways, together called the microbiome–gut–brain axis (30–34). In general, communication between microbiota and the brain is divided into four categories: the neuronal route (enteric nervous system and vagus nerve), the endocrine route (*e.g.* cortisol), the metabolic route (*e.g.* short chain fatty acids (SCFAs) and tryptophan), and the immunological route (*e.g.* cytokines and immune cells) (35, 36). Bacteria can also affect the composition of the gut microbiota, thereby indirectly affecting gut-brain signaling [Cryan and Dinan (35)].

## *Helicobacter pylori* and Neurological Disorders

A gastric spiral-shaped, Gram-negative microorganism, called *H. pylori*, colonizes the stomach of more than half of the world’s human population albeit with large geographical variations. Next to gastritis, peptic ulcer disease, mucosa-associated lymphoid tissue (MALT) -lymphoma, and adenocarcinoma, *H. pylori* infection has also been associated with neurological diseases.

**Figure 1:**
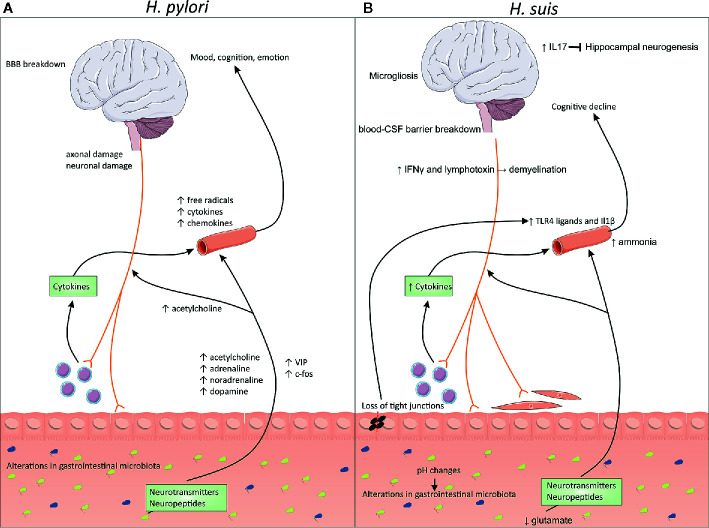
Changes at the microbiome–gut–brain axis during *Helicobacter pylori* and Non-*H. pylori Helicobacter* (NHPH) infection. **(A)** *H. pylori* is associated with persistent local inflammation, which might lead to systemic inflammation, characterized by increased levels of free radicals, cytokines and chemokines in the blood. Infection also leads to the release of several neurotransmitters, such as acetylcholine, adrenaline, noradrenaline and dopamine, but also increased levels of neuropeptides, such as vasoactive intestinal peptide (VIP) and c-fos. Moreover, *H. pylori* can lead to blood–brain barrier breakdown and axonal/neuronal damage. **(B)** *H. suis* is associated with inflammation of the stomach, associated with loss of the gastrointestinal barrier function, leading to leakage of TLR4 ligands into the blood. This leads to the breakdown of the blood-CSF barrier, combined with microgliosis and cognitive decline. *H. suis-*induced changes in the pH possibly leads to changes in the gastrointestinal microbiome. Moreover, lower levels of glutamate are present, which could influence the production of several neurotransmitters. Higher levels of IL-17 can block hippocampal neurogenesis while IFN-γ and lymphotoxins could lead to demyelination. BBB, blood–brain barrier; CSF, cerebrospinal fluid; IL, interleukin; IFN, interferon.

Even though both innate and acquired immune responses are activated in individuals infected with *H. pylori*, the host is unable to eradicate the bacteria, leading to a chronic lifelong infection (37, 38). To escape the host’s immune response and to survive in the hostile conditions found in the stomach, *H. pylori* has developed several strategies, including manipulating innate immune receptors and inhibiting effector T-cell responses (39, 40). The mechanism to evade the immune system depends on the presence or absence of certain bacterial virulence factors (39). The evoked immune response by the host can lead to the local secretion of various inflammatory mediators, such as interleukin (IL) 8, -6, -1β, -10, and -12, tumor necrosis factor (TNF) and interferon (IFN) γ, which might reach the circulation causing a systemic effect (41, 42). The persistence of noticeable local and systemic concentrations of these pro-inflammatory factors can induce neuroinflammation and -toxicity (41). Next to this, *H. pylori* infection leads to the release of several neurotransmitters, such as acetylcholine, adrenaline, noradrenaline, serotonin, and dopamine (43, 44). Moreover, *H. pylori* infection might lead to axonal/neuronal damage, production of free radicals, and changes in neuropeptide expression, such as vasoactive intestinal peptide (VIP) and c-fos (43). Lastly, *H. pylori* infection is associated with changes in the composition of the gastrointestinal microbiome (43, 45). These changes, illustrated in **Figure 1A**, can potentially alter the outcome of neurological disorders.

Indeed, seropositivity for *H. pylori* has been associated with poor cognition (46), neurologic impairment (47), and cerebrovascular disease (48) and is recognized as a significant risk factor for the development of dementia (21, 49). Next to an association of *H. pylori* with Parkinson’s disease (50), it has also been shown that infection with *H. pylori* increases the risk of developing Parkinson’s disease (41, 51, 52). Moreover, it has been shown that eradication of *H. pylori* improves the motor symptoms associated with Parkinson’s disease (53, 54). Interestingly, *H. pylori* might influence the bioavailability of L-3,4-dihydroxyphenylalaline (L-DOPA), the most common treatment for Parkinson’s disease (52, 55).

*H. pylori* might also play a role in Alzheimer’s disease as discussed in a review by Doulberis et al. (56). *H. pylori* infection is associated with mild cognitive impairment, a prodromal phase of Alzheimer’s disease (57, 58) and with Alzheimer’s disease itself (59). Higher levels of neuro-inflammation have been found in Alzheimer’s disease patients infected with *H. pylori*, which correlated with cognitive decline (60, 61), whereas eradication of *H. pylori* improved the cognitive and functional abilities (62, 63).

In multiple sclerosis (MS), however, *H. pylori* is found less in patients compared to control ones (64) and infection is even thought to be beneficial (65). Lower clinical signs were found in mice infected with *H. pylori* compared to control animals (66).

## There is More in the Stomach Than *Helicobacter pylori*

Since the description of *H. pylori*, many other gastric species in the genus *Helicobacter* have been described. These gastric non-*H. pylori Helicobacter* (NHPH) species have been reported in the stomach of various hosts, including pigs, dogs, cats, and non-human primates and some of them have a zoonotic potential (67, 68). The most prevalent gastric NHPH species in humans is *Helicobacter suis* which naturally colonizes the stomach of pigs and non-human primates (67, 68). The bacterium is of zoonotic importance, infecting 0.2–6% of the human population, causing gastritis, peptic ulcers, and MALT lymphoma (67). However, since some infections with this microorganism remain subclinical, their true prevalence in humans is probably underestimated (67). Furthermore, these spiral-shaped bacteria are not always found in the human stomach after investigation of a small biopsy sample due to their focal and patchy colonization pattern (67, 69–71). Like *H. pylori*, *H. suis* may lead to a life-long infection, associated with a tolerogenic immune response (24, 72).

In literature, hardly any data is available on the association between an infection with NHPH species and neurological disorders. Indeed, there are no papers describing the association of NHPH with neurodegenerative or -immunological disorders like amyotrophic lateral sclerosis, spinocerebellar degeneration, acute disseminated encephalomyelitis, and Guillain-Barré syndrome. One study showed that mice infected with *Helicobacter felis* display both gastric and neuroinflammation (73). In another study, a remarkable high presence of *H. suis* DNA (27%) was found in gastric biopsies from idiopathic Parkinson’s disease patients compared to a control group without clinical symptoms of Parkinson’s disease (2%) (74). This was not the case for other zoonotically important gastric NHPH species. Additionally, *H. suis* DNA was found in a blood sample of a patient simultaneously affected by Parkinson’s and Alzheimer’s disease. After eradication of the *H. suis* infection, the patient’s gastric and neurological symptoms improved remarkably (74). Moreover, *H. suis* infection in Parkinson’s patients has recently been linked with higher mortality (75). To our knowledge, there are no other papers describing a role for *H. suis* in neurological disorders. Here, we will discuss several possible ways *H. suis* might influence the brain. These changes are summarized in **Figure 1B**.

## *Helicobacter suis* and the Microbiome–Gut–Brain Axis

In the first part, inflammatory changes in the stomach and how they might affect the brain *via* the systemic circulation are discussed. In the second part, changes due to virulence factors of *H. suis* and the effect on the microbiome are discussed.

### Inflammatory Changes and Gastrointestinal Barrier Functioning

Infection with *H. suis* in pigs and mice is associated with increased inflammation in the stomach, characterized by the higher expression of IL-8, -10, -1β, and -4, keratinocyte chemoattractant (KC), lipopolysaccharide-induced CXC chemokine (LIX), and macrophage inflammatory protein (MIP2) depending on the host (72, 76–78). This leads to the infiltration of B- and T-cells and macrophages in mice, inducing a Th2 response.

Gastritis is accompanied by mucosal edema (67) and gastric epithelial cell death (79), all of which could compromise the integrity of the gastrointestinal barrier. The gastrointestinal barrier consists of two layers: the epithelial cell layer, connected by tight junctions, and a mucus layer. In pigs, significant downregulation of claudin 18 (CLDN18) was found in the stomach of *H. suis* infected animals (72). In a recent mouse study, we found increased permeability of the gastrointestinal barrier after *H. suis* infection, accompanied by increased expression of mucine 13 (Muc13) and aberrant localization of zonula occludens 1 (ZO1) (77). This further progressed to systemic inflammation, characterized by the leakage of TLR4 ligands into the blood, affecting the brain homeostasis *via* the blood–cerebrospinal fluid barrier (77). Next to TLR4 ligands, also IL1β was found in the serum of *H. suis-*infected mice, which is shown to induce inflammatory gene expression in the hippocampus and hypothalamus associated with sickness behavior (80). As discussed below, also other molecules that are observed in the stomach upon *H. suis* infection might affect the brain when reaching the systemic circulation due to a leaky gut.

Next to the Th2 response, also a Th17 response has been associated with *H. suis* infection in the different hosts (mice, gerbils, pigs, and humans), characterized by the presence of Th17 cells and/or increased levels of IL-17 in the stomach (76, 78, 81, 82). IL-17 is known to block adult hippocampus neurogenesis (83) and is linked to depression in MS (84). In gerbils, but not mice, also increased levels of IFN-γ were found in the stomach of *H. suis* infected animals (81). IFN-γ is shown to be a regulator of the neural precursor pool in the non-inflamed brain (85) but is also linked with demyelination due to the reduced proliferation and viability of oligodendroglial cells (86, 87).

*H. suis* is also associated with increased levels of lymphotoxin (LT)-α and -β in the stomach of mice (88). These cytokines are not only involved in the generation of follicular dendritic cells (89), but also regulate neuronal and glial lineage differentiation (90). Lymphotoxins have been shown to play a role in MS, causing demyelination due to oligodendrocyte toxicity (91). Blocking lymphotoxin in experimental autoimmune encephalomyelitis (EAE), a mouse model of MS, reduces disease symptoms, which is accompanied with lower levels of the chemokine CXCL13 (92). This chemokine plays a role in the recruitment of B-cells and its expression is increased in the stomach after *H. suis* infection in both pigs, mice, and gerbils (72, 81), as are other chemokines such as C-X-C motif chemokine receptor (CXCR) 7, 15 and 4, C-C motif chemokine ligand (CCL) 19 and 21, and C-X-C motif chemokine ligand 12 (CXCL12) (88). In MS, higher levels of CXCL13 have been observed in B-cell aggregates in the inflamed meninges (92) and correlate with demyelination, neural cell loss, and rapid disease progression (93). Thus, higher levels of CXCL13 caused by a *H. suis* infection can potentially lead to accelerated disease progression.

### Changes Due to Virulence Factors, Metabolism and Microbiome

*H. suis* affects the presence of glutamine and glutathione by its virulence factor γ-glutamyl transpeptidase (GGT), in this way damaging epithelial cells (81, 82, 94). Glutamine and glutathione are not only important for the health of gastrointestinal tissue (95), they are also precursors for the neurotransmitters glutamate, aspartate, and γ-amino butyric acid (GABA), which are important neurotransmitters. Depletion of glutamine, caused by *H. suis* infection, could thus lead to changes in these neurotransmitters, affecting gut–brain signaling.

Urea is converted by *H. suis* to ammonia by the presence of urease (96, 97). High levels of ammonia are linked to encephalopathy, associated with neuropsychiatric and neurological symptoms (98, 99). Although it is unlikely that an *H. suis* infection leads to high levels of ammonia, the continuous exposure of slightly higher levels could also interfere with normal brain functioning.

Parietal cells are also affected by *H. suis*-associated inflammation. This leads to changes in the expression and functioning of H^+^/K^+^-ATPase and subsequent changes in pH, which is associated with more fluid gastric content (72). These changes can subsequently influence the gastric microbiota. Indeed, more *Fusobacterium gastrosuis* was found in *H. suis* infected pigs (100). Infection with *H. felis*, another NHPH known to infect humans, is associated with a decrease in *Lactobacillus* and an increase in *Clostridium*, *Bacteroidetes*, *Prevotella*, *Eubacterium*, *Ruminococcus*, *Streptococcus*, and *E. coli* in the stomach (94, 101). *Lactobacillus* has been shown to secrete acetylcholine, which is important in regulating memory, attention, and learning, and has therapeutic effects in mental illnesses, reducing anxiety and depression (102). Lower numbers of *Lactobacillus* due to *H. suis* could thus possibly affect mood. Increased levels of *Clostridium* has been linked to autism (103), indicating that increased presence of *Clostridium* in *H. suis-*infected animals might affect brain homeostasis.

## Conclusion

Numerous studies have been published about the possible effect of a *H. pylori* infection on neurological diseases, while other *Helicobacter* species have hardly been studied. However, recent studies report on a possible link between *H. suis* infection and Parkinson’s disease. Here, we describe several possible pathways in the microbiome–gut–brain axis which could be influenced by *H. suis* infection. Altogether, this highlights the importance of gaining more insights in the role of *non-Helicobacter pylori Helicobacter* species in neurological diseases.

## Author Contributions

NG wrote the manuscript. EV, RV, AS, and FH advised and reviewed the manuscript. All authors contributed to the article and approved the submitted version.

## Funding

NG is supported by FWO Vlaanderen. Research in the author’s lab is sponsored by FWO Vlaanderen, Ghent University, VIB, and the Baillet Latour Fund.

## Conflict of Interest

The authors declare that the research was conducted in the absence of any commercial or financial relationships that could be construed as a potential conflict of interest.
